# Supplementary material for: Resolving the full spectrum of human genome variation using Linked-Reads
Source: Genome Res. 2019 Apr;29(4):635–45. doi: 10.1101/gr.234443.118 (PMC6442396; doi:10.1101/gr.234443.118)
Supplement: Supplemental Material [file supp_gr.234443.118_Supplemental_longranger-2.2.zip › longranger-2.2.tar.bz2!/lr2.2-public/lib/stan/cmdstan/stan/doc/api/html/print_8hpp_source.html]

Stan: src/stan/command/print.hpp Source File


|  |  |
| --- | --- |
|  | Stan  2.5.0  probability, sampling & optimization |


- Main Page
- Related Pages
- Namespaces
- Classes
- Files

- File List
- File Members

All Classes Namespaces Files Functions Variables Typedefs Enumerations Enumerator Friends Macros Pages

- src
- stan
- command

print.hpp

Go to the documentation of this file.

1 #ifndef STAN\_\_COMMAND\_\_PRINT\_HPP

2 #define STAN\_\_COMMAND\_\_PRINT\_HPP

3

4 #include <algorithm>

5 #include <iostream>

6 #include <iomanip>

7 #include <ios>

8 #include <stan/mcmc/chains.hpp>

9

10 void compute\_width\_and\_precision(double value, int sig\_figs, int& width, int& precision) {

11

12  double abs\_value = std::fabs(value);

13

14  if (value == 0) {

15  width = sig\_figs;

16  precision = sig\_figs;

17  }

18  else if (abs\_value >= 1) {

19  int int\_part = std::ceil(log10(abs\_value) + 1e-6);

20  width = int\_part >= sig\_figs ? int\_part : sig\_figs + 1;

21  precision = int\_part >= sig\_figs ? 0 : sig\_figs - int\_part;

22  }

23  else {

24  int frac\_part = std::fabs(std::floor(log10(abs\_value)));

25  width = 1 + frac\_part + sig\_figs;

26  precision = frac\_part + sig\_figs - 1;

27  }

28

29  if (value < 0) ++width;

30

31 }

32

33 int compute\_width(double value, int sig\_figs) {

34  int width;

35  int precision;

36  compute\_width\_and\_precision(value, sig\_figs, width, precision);

37  return width;

38 }

39

40 int compute\_precision(double value, int sig\_figs, bool scientific) {

41

42  if (scientific) {

43  return sig\_figs - 1;

44  }

45  else {

46  int width;

47  int precision;

48  compute\_width\_and\_precision(value, sig\_figs, width, precision);

49  return precision;

50  }

51

52 }

53

54 int calculate\_column\_width(const Eigen::VectorXd& x,

55  const std::string& name,

56  const int sig\_figs,

57  std::ios\_base::fmtflags& format) {

58

59  int padding = 2;

60

61  // Fixed Precision

62  size\_t fixed\_threshold = 8;

63  size\_t max\_fixed\_width = 0;

64

65  for (int i = 0; i < x.size(); ++i) {

66  size\_t width = compute\_width(x[i], sig\_figs);

67  max\_fixed\_width = width > max\_fixed\_width ? width : max\_fixed\_width;

68  }

69

70  if (max\_fixed\_width + padding < fixed\_threshold) {

71  format = std::ios\_base::fixed;

72  max\_fixed\_width = name.length() > max\_fixed\_width ? name.length() : max\_fixed\_width;

73  return max\_fixed\_width + padding;

74  }

75

76  // Scientific Notation

77  size\_t scientific\_width = sig\_figs + 1 + 4; // Decimal place + exponent

78  if (x.minCoeff() < 0) ++scientific\_width;

79

80  scientific\_width = name.length() > scientific\_width ? name.length() : scientific\_width;

81

82  format = std::ios\_base::scientific;

83  return scientific\_width + padding;

84

85 }

86

87 Eigen::VectorXi calculate\_column\_widths(const Eigen::MatrixXd& values,

88  const Eigen::Matrix<std::string, Eigen::Dynamic, 1>& headers,

89  const int sig\_figs,

90  Eigen::Matrix<std::ios\_base::fmtflags, Eigen::Dynamic, 1>& formats) {

91  int n = values.cols();

92  Eigen::VectorXi column\_widths(n);

93  formats.resize(n);

94  for (int i = 0; i < n; i++) {

95  column\_widths(i) = calculate\_column\_width(values.col(i), headers(i), sig\_figs, formats(i));

96  }

97  return column\_widths;

98 }

99

100 void print\_usage() {

101

102  std::cout << "USAGE: print <filename 1> [<filename 2> ... <filename N>]"

103  << std::endl

104  << std::endl;

105

106  std::cout << "OPTIONS:" << std::endl << std::endl;

107  std::cout << " --autocorr=<chain\_index>\tAppend the autocorrelations for the given chain"

108  << std::endl

109  << std::endl;

110  std::cout << " --sig\_figs=<int>\tSet significant figures of output (Defaults to 2)"

111  << std::endl

112  << std::endl;

113

114 }

115

116 bool is\_matrix(const std::string& parameter\_name) {

117  return (parameter\_name.find("[") != std::string::npos);

118 }

119

120 std::string base\_param\_name(stan::mcmc::chains<>& chains, const int index) {

121  std::string name = chains.param\_name(index);

122  return name.substr(0, name.find("["));

123 }

124

125 std::string matrix\_index(stan::mcmc::chains<>& chains, const int index) {

126  std::string name = chains.param\_name(index);

127  return name.substr(name.find("["));

128 }

129

130 std::vector<int> dimensions(stan::mcmc::chains<>& chains, const int start\_index) {

131  std::vector<int> dims;

132  int dim;

133

134  std::string name = base\_param\_name(chains, start\_index);

135  int last\_matrix\_element = start\_index;

136  while (last\_matrix\_element+1 < chains.num\_params()) {

137  if (base\_param\_name(chains, last\_matrix\_element+1) == name)

138  last\_matrix\_element++;

139  else

140  break;

141  }

142

143  std::stringstream ss(matrix\_index(chains, last\_matrix\_element));

144  ss.get();

145  ss >> dim;

146

147  dims.push\_back(dim);

148  while (ss.get() == ',') {

149  ss >> dim;

150  dims.push\_back(dim);

151  }

152  return dims;

153 }

154

155 // next 1-based index in row major order

156 void next\_index(std::vector<int>& index, const std::vector<int>& dims) {

157  if (dims.size() != index.size())

158  throw std::domain\_error("next\_index: size mismatch");

159  if (dims.size() == 0)

160  return;

161  index[index.size()-1]++;

162

163  for (int i = index.size()-1; i > 0; i--) {

164  if (index[i] > dims[i]) {

165  index[i-1]++;

166  index[i] = 1;

167  }

168  }

169

170  for (size\_t n = 0; n < dims.size(); n++) {

171  if (index[n] <= 0 || index[n] > dims[n]) {

172  std::stringstream message\_stream("");

173  message\_stream << "next\_index: index[" << n << "] out of bounds. "

174  << "dims[" << n << "] = " << dims[n] << "; "

175  << "index[" << n << "] = " << index[n];

176  throw std::domain\_error(message\_stream.str());

177  }

178  }

179 }

180

181 // return the flat 0-based index of a column major order matrix based on the

182 // 1-based index

183 int matrix\_index(std::vector<int>& index, const std::vector<int>& dims) {

184  if (dims.size() != index.size())

185  throw std::domain\_error("next\_index: size mismatch");

186  if (dims.size() == 0)

187  return 0;

188  for (size\_t n = 0; n < dims.size(); n++) {

189  if (index[n] <= 0 || index[n] > dims[n]) {

190  std::stringstream message\_stream("");

191  message\_stream << "matrix\_index: index[" << n << "] out of bounds. "

192  << "dims[" << n << "] = " << dims[n] << "; "

193  << "index[" << n << "] = " << index[n];

194  throw std::domain\_error(message\_stream.str());

195  }

196  }

197

198  int offset = 0;

199  int prod = 1;

200  for (size\_t i = 0; i < dims.size(); i++) {

201  offset += (index[i]-1) \* prod;

202  prod \*= dims[i];

203  }

204  return offset;

205 }

206

207 #endif

chains.hpp

stan::agrad::fabs

fvar< T > fabs(const fvar< T > &x)

**Definition:** fabs.hpp:18

stan::mcmc::chains::param\_name

const std::string & param\_name(int j) const

**Definition:** chains.hpp:352

dimensions

std::vector< int > dimensions(stan::mcmc::chains<> &chains, const int start\_index)

**Definition:** print.hpp:130

compute\_width\_and\_precision

void compute\_width\_and\_precision(double value, int sig\_figs, int &width, int &precision)

**Definition:** print.hpp:10

stan::agrad::floor

fvar< T > floor(const fvar< T > &x)

**Definition:** floor.hpp:15

stan::agrad::ceil

fvar< T > ceil(const fvar< T > &x)

**Definition:** ceil.hpp:15

stan::math::dims

void dims(const T &x, std::vector< int > &result)

**Definition:** dims.hpp:13

matrix\_index

std::string matrix\_index(stan::mcmc::chains<> &chains, const int index)

**Definition:** print.hpp:125

calculate\_column\_widths

Eigen::VectorXi calculate\_column\_widths(const Eigen::MatrixXd &values, const Eigen::Matrix< std::string, Eigen::Dynamic, 1 > &headers, const int sig\_figs, Eigen::Matrix< std::ios\_base::fmtflags, Eigen::Dynamic, 1 > &formats)

**Definition:** print.hpp:87

compute\_precision

int compute\_precision(double value, int sig\_figs, bool scientific)

**Definition:** print.hpp:40

stan::math::prod

T prod(const std::vector< T > &v)

Returns the product of the coefficients of the specified standard vector.

**Definition:** prod.hpp:17

stan::mcmc::chains

An mcmc::chains object stores parameter names and dimensionalities along with samples from multiple c...

**Definition:** chains.hpp:55

stan::mcmc::chains::num\_params

int num\_params() const

**Definition:** chains.hpp:344

base\_param\_name

std::string base\_param\_name(stan::mcmc::chains<> &chains, const int index)

**Definition:** print.hpp:120

stan::math::e

double e()

Return the base of the natural logarithm.

**Definition:** constants.hpp:86

compute\_width

int compute\_width(double value, int sig\_figs)

**Definition:** print.hpp:33

is\_matrix

bool is\_matrix(const std::string &parameter\_name)

**Definition:** print.hpp:116

calculate\_column\_width

int calculate\_column\_width(const Eigen::VectorXd &x, const std::string &name, const int sig\_figs, std::ios\_base::fmtflags &format)

**Definition:** print.hpp:54

next\_index

void next\_index(std::vector< int > &index, const std::vector< int > &dims)

**Definition:** print.hpp:156

print\_usage

void print\_usage()

**Definition:** print.hpp:100

stan::agrad::log10

fvar< T > log10(const fvar< T > &x)

**Definition:** log10.hpp:15


---

[ Stan Home Page ]

*© 2011–2014,
Stan Development Team.*
